# Inertial cavitation detection during in-vitro sonothrombolysis

**DOI:** 10.1186/2050-5736-2-S1-A12

**Published:** 2014-12-10

**Authors:** Antonella Verbeni, Andrea Cafarelli, Piero Miloro, Arianna Menciassi

**Affiliations:** 1 The BioRobotics Institute, Scuola Superiore Sant’Anna, Pisa, 56025, Italy

## Background

Being cardiovascular diseases the leading cause of death worldwide, a prompt intervention is needed to restore blood flow. Sonothrombolysis with/without addiction of thrombolytic drugs is explored as a promising solution, thanks to its non-invasiveness, precision and action quickness. Even if the involved mechanisms are not completely understood, acoustic cavitation seems to play a significant role [1]. Several efforts are currently devoted to optimizing sonication parameters [2]. A thorough investigation on the involved physical phenomena is expected to further speed-up the process.

## Materials and methods

The experimental setup is composed of a Passive Cavitation Detector (PCD) recording pressure fluctuations of oscillating bubbles and mounted confocally to a 1MHz High Intensity Focused Ultrasound (HIFU) transducer. Confocality has been verified by mapping the pressure fields of both devices with a 0.2mm needle hydrophone.

**Figure 1:**
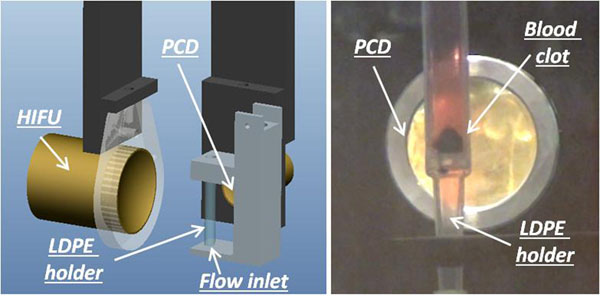
CAD and picture of the experimental setup.

An LDPE holder containing the thrombus is placed at the foci (Figure 1) through a 3 axis positioning frame. A constant flow of tap water (2ml/min) is established.

To detect inertial cavitation (broadband emission and increase in white noise), the PCD signal has been filtered analogically at 5MHz to remove harmonic frequencies which could saturate the acquisition system.

## Results

In-vitro sonothrombolysis tests have been performed on human blood clots exposed for two minutes to an acoustic field acoustic field (65W power, 25mm focal length, 3mm focal diameter, 450μs pulse length, 1:10 duty cycle). The same parameters were previously credited to avoid thermal damage [3].

**Figure 2:**
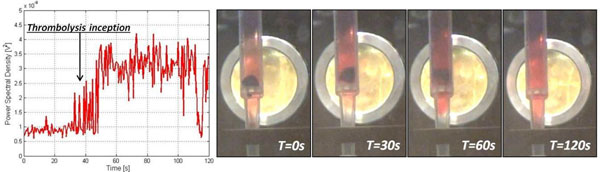
Power spectral density of the signal acquired by the PCD (left) and screenshots from a synchronized video during the thrombolysis test (right).

During the test, PCD signal was digitalized twice per second, at a sample frequency of 40MHz, synchronized with the HIFU burst. Power spectral density was calculated in the 5-12MHz band, with digital notch filters at the super-harmonic frequencies, in order to quantify the cavitation dose. Figure 2 shows the results of a sonothrombolysis test. A temporal correlation between thrombolysis inception and white noise increase can be observed.

## Conclusion

The proposed setup demonstrated the ability to detect inertial cavitation during in-vitro sonothrombolysis tests; a correlation between thrombolysis inception and white noise increase was found. Statistically significant analysis will be performed to verify this correlation, thus allowing the optimization of sonothrombolysis parameters and protocols to enhance cavitational effects.
